# MALDI-TOF mass spectrometry-based serotyping of *V. parahaemolyticus* isolated from the Zhejiang province of China

**DOI:** 10.1186/s12866-018-1328-z

**Published:** 2018-11-13

**Authors:** Ping Li, Wenwen Xin, Susu Xia, Yun Luo, Zhongwen Chen, Dazhi Jin, Shan Gao, Hao Yang, Bin Ji, Henghui Wang, Yong Yan, Lin Kang, Jinglin Wang

**Affiliations:** 1 Graduate College, Anhui Medical University, No.81 Meishan Road, Hefei, 230032 Anhui China; 2 Jiaxing Key Laboratory of Pathogenic Microbiology, Jiaxing Center for Disease Control and Prevention, No.486 Wenqiao Road, Nanhu district, Jiaxing, 314050 China; 3 State Key Laboratory of Pathogen and Biosecurity, Beijing Institute of Microbiology and Epidemiology, No.20 Dongdajie, Fengtai, Beijing, 100071 China; 4 Department of Microbiology, Zhejiang Province Center for Disease Control and Prevention, No.3399 Binsheng Road, Hangzhou, 310000 China

**Keywords:** *Vibrio parahaemolyticus*, MALDI-TOF MS, O4:K8, Biomarkers

## Abstract

**Background:**

*Vibrio parahaemolyticus* is as an important food-borne pathogen circulating in China. Since 1996, the core serotype has become O3:K6, which has specific genetic markers. This serotype causes the majority of outbreaks worldwide. Until now, nearly 21 serotypes were considered as serovariants of O3:K6. Among these, O4:K68, O1:K25 and O1:KUT have caused pandemic outbreaks. O4:K8, a serovariant of O3:K6, has become the second most dominant serotype circulating in China after O3:K6. In this study, we report the use of matrix-assisted laser desorption/ionization time-of-flight mass spectrometry (MALDI-TOF MS) to analyze and characterize 146 *V. parahaemolyticus* isolates belonging to 23 serotypes.

**Results:**

Upon mass spectral analysis, isolates belonging to O4:K8 formed a distinct group among the five main pandemic groups (O3:K6, O4:K8, O4:K68, O1:K25 and O1:KUT). Two major protein peaks (*m/z* 4383 and 4397) were significantly different between serotype O4:K8 and the four other pandemic strains. Both of these peaks were present in 32 out of 36 O4:K8 isolates, but were absent in 105 out of 110 non-O4:K8 isolates. These peaks were also absent in all 74 pandemic serotypes (O3:K6, O4:K68, O1:K25 and O1:KUT).

**Conclusion:**

Our results highlight the threat of O4:K8 forming a distinct group, which differs significantly from pandemic serotypes on the proteomic level. The use of MALDI-TOF MS has not been reported before in a study of this nature. Mass spectrum peaks at *m/z* 4383 and 4397 may be specific for O4:K8. However, we cannot conclude that MALDI-TOF MS can be used to serotype *V. parahaemolyticus*.

**Electronic supplementary material:**

The online version of this article (10.1186/s12866-018-1328-z) contains supplementary material, which is available to authorized users.

## Background

*Vibrio parahaemolyticus*, a curved, Gram-negative bacterium that inhabits estuarine and marine environments, is a major cause of foodborne illness worldwide and is one of the leading causes of foodborne illness in South China [1, 2]. It was first identified in Japan during an outbreak in the 1950s, which caused 272 illnesses. *V. parahaemolyticus* infections are mainly associated with the consumption of contaminated raw or undercooked seafood and may lead to acute gastroenteritis, wound infection and septicemia [3]. Virulence factors associated with the *V. parahaemolyticus* pathogen include thermostable direct haemolysin (TDH), TDH-related haemolysin (TRH) and the two type III secretion systems [4] T3SS1 [5] and T3SS2. TDH and TRH may have hemolytic and cytotoxicity activity in the host cell [6, 7]. T3SS1 is found in all *V. parahaemolyticus* strains, whereas T3SS2 is found in clinical strains and is associated with pandemic strains.

*V. parahaemolyticus* may respond to environmental changes and human immune responses by the serovar alteration of its somatic (O) and capsular (K) antigen-encoding genes. This can occur via mutation or horizontal gene transfer. The O- and K antigen-encoding loci are adjacent to each other, and therefore the O- and K-antigens may be simultaneously mutated through a single recombination event [8]. Until now, 13 O serogroups and 71 K serotypes have been identified based on the antigenic properties of their O and K antigens [9–11]. The O3:K6 serotype is mainly associated with pandemics, however the serotypes O4:K68, O1:K25 and O1:KUT, which are regarded as serovariants of O3:K6, have also been associated with worldwide outbreaks. For instance, O3:K6 and O1:KUT were the main serotypes associated with the 2008–2014 outbreaks in South China. The number of infections caused by the O4:K8 serotype have been increasing in recent years [12]. The O4:K8 virulence factors are the same as those of the O3:K6 pandemic clones, but are genetically distinct from those of the O3:K6, O1:KUT and other internationals strains [13].

Thiosulfate-citrate-bile salts-sucrose (TCBS) is an agar that is used to isolate and identify *Vibrios* species, including *V. parahaemolyticus.* Typically, colonies of *V. parahaemolyticus* appear opaque, round, 2–3 mm in diameter and green or bluish in color [14]. Molecular methods may also be used to identify this species. For example, a polymerase chain reaction (PCR) method that targets the *toxR* gene can identify *V. parahaemolyticus* strains at the species level [15]. Multiplex PCR methods that amplify the *tdh*, *trh* and *tlh* genes can detect all strains of *V. parahaemolyticus* [16]*.* Real-time PCR and loop mediated isothermal amplification (LAMP) [17] have also been used for the identification of *V. parahaemolyticus*. Finally, MALDI-TOF MS has been used to identify *V. parahaemolyticus* in clinical diagnosis [18].

Many molecular typing methods have been implemented in *V. parahaemolyticus* subtyping. The principal methods are pulsed-field gel electrophoresis (PFGE) [19], which can provide genetic diversity not shown in group specific PCR (GS-PCR), and multilocus sequence typing (MLST) [20], which is a typing method based on seven housekeeping genes. Subtyping based on protein profiles, such as MALDI-TOF MS, has also been used to differentiate environment strains [21].

MALDI-TOF MS is a useful tool in routine clinical diagnosis, because it has the ability to rapidly identify bacterial strains with high accuracy. Recently, several research groups have applied MALDI-TOF MS to the serotyping of *Salmonella enterica subsp. Enterica* [22], *Vibrio cholerae* [23] and *Escherichia coli* [24]. In addition, Kang et al. attempted to subtype *Salmonella enterica* serovar Typhimurium using MALDI-TOF MS [25] but failure. However, there have been few reports on the application of MALDI-TOF MS to the serotyping of *V. parahaemolyticus*. In this study, we evaluated the ability of MALDI-TOF MS to screen 23 serotypes from a collection of 146 strains of *V. parahaemolyticus* isolated in the Zhejiang province of China.

## Results

### Identification of *V. parahaemolyticus* by *toxR*-targeted PCR and MALDI-TOF MS

All 146 strains (Table 1) in this study were *toxR*^*+*^ and identified at the species level. For MALDI-TOF MS identification, the spectra of all strains were transferred into BioTyper 4.0 software and compared with the reference database supplied by the manufacturer. All strains were identified at the species level (data not shown).

**Table 1:** *Vibrio parahaemolyticus* strains used in this study

Serovar	Sources	Years	No. of strains
O3:K6	Zhejiang, China	2009, 2010, 2011, 2012	63
O4:K8	Zhejiang, China	2009, 2010, 2011, 2012	36
O4:K68	Zhejiang, China	2011, 2012	4
O1:K25	Zhejiang, China	2011	2
O1:KUT	Zhejiang, China	2009, 2011, 2012	5
O11:KUT	Zhejiang, China	2011	2
O8:K41	Zhejiang, China	2011, 2012	7
O1:K36	Zhejiang, China	2012, 2010	4
O2:KUT	Zhejiang, China	2011,	1
O4:K42	Zhejiang, China	2012	2
O4:KUT	Zhejiang, China	2011, 2012	2
O5:K68	Zhejiang, China	2011	3
O2:K3	Zhejiang, China	2012	1
O4:K13	Zhejiang, China	2011	1
O4:K9	Zhejiang, China	2012	2
O1:K68	Zhejiang, China	2012	1
O1:K8	Zhejiang, China	2009	1
O10:K60	Zhejiang, China	2011	1
O11:K50	Zhejiang, China	2011	1
O2:K22	Zhejiang, China	2012	1
O3:K29	Zhejiang, China	2011	1
O3:K36	Zhejiang, China	2009	1
O3:K56	Zhejiang, China	2012	1
O3:K68	Zhejiang, China	2012	1
O5:K15	Zhejiang, China	2012	1
O6:K18	Zhejiang, China	2011	1

A total of 35 randomly selected strains, representing five main pandemic serotypes, were used to establish a serotype-specific reference database. The spectral quality of each strain was evaluated in terms of the presence and intensity of various peaks using Flex analysis software. Low-quality spectra were replaced with spectra obtained from the fresh spotting of the same protein extract. Comparisons were also performed between the original commercial database and the expanded database that contained our in-house entries. All strains that were not used in database construction received higher scores from the expanded database containing in-house entries than from the manufacturer’s reference database (Table 2).

**Table 2:** Comparison of identification by commercial database and expanded database after introduction our in-house entries

Strain ID	Serotype	Log (score)	
		Commercial database	Expanded database
030	O3:K6	2.394	2.703
072	O3:K6	2.379	2.755
078	O3:K6	2.325	2.8
099	O3:K6	2.386	2.822
100	O3:K6	2.346	2.722
101	O3:K6	2.435	2.798
104	O3:K6	2.317	2.739
105	O3:K6	2.437	2.806
108	O3:K6	2.476	2.808
115	O3:K6	2.386	2.746
116	O3:K6	2.386	2.706
119	O3:K6	2.399	2.746
123	O3:K6	2.385	2.737
126	O3:K6	2.384	2.671
128	O3:K6	2.345	2.612
136	O3:K6	2.384	2.783
139	O3:K6	2.436	2.7
140	O3:K6	2.236	2.68
147	O3:K6	2.435	2.795
152	O3:K6	2.448	2.715
153	O3:K6	2.462	2.765
161	O3:K6	2.436	2.767
162	O3:K6	2.446	2.714
170	O3:K6	2.401	2.783
176	O3:K6	2.45	2.68
177	O3:K6	2.418	2.78
178	O3:K6	2.476	2.777
179	O3:K6	2.44	2.754
183	O3:K6	2.364	2.778
184	O3:K6	2.396	2.619
189	O3:K6	2.358	2.777
190	O3:K6	2.439	2.79
191	O3:K6	2.421	2.796
192	O3:K6	2.46	2.734
194	O3:K6	2.406	2.749
195	O3:K6	2.427	2.79
197	O3:K6	2.454	2.795
199	O3:K6	2.454	2.781
207	O3:K6	2.403	2.725
240	O3:K6	2.422	2.756
241	O3:K6	2.379	2.751
243	O3:K6	2.439	2.747
245	O3:K6	2.396	2.712
013	O1:KUT	2.307	2.524
102	O1:KUT	2.304	2.571
124	O1:KUT	2.319	2.792
021	O1:K25	2.168	2.626
006	O4:K68	2.337	2.734
026	O4:K68	2.455	2.769
059	O4:K8	2.43	2.751
060	O4:K8	2.357	2.787
064	O4:K8	2.338	2.807
076	O4:K8	2.398	2.793
079	O4:K8	2.466	2.787
081	O4:K8	2.455	2.808
084	O4:K8	2.31	2.747
098	O4:K8	2.358	2.762
107	O4:K8	2.405	2.799
110	O4:K8	2.412	2.8
117	O4:K8	2.322	2.706
122	O4:K8	2.387	2.722
125	O4:K8	2.43	2.729
129	O4:K8	2.394	2.844
130	O4:K8	2.427	2.73
132	O4:K8	2.359	2.778
135	O4:K8	2.39	2.709
137	O4:K8	2.348	2.824
141	O4:K8	2.368	2.771
155	O4:K8	2.35	2.806
167	O4:K8	2.403	2.695
173	O4:K8	2.44	2.629
174	O4:K8	2.356	2.766
182	O4:K8	2.439	2.793
187	O4:K8	2.276	2.652
242	O4:K8	2.306	2.745

### Phyloproteomic analysis of 23 serotypes

The results of a cluster analysis of 146 strains of 23 serotypes are shown in Additional file 1: Figure S1. The strains were separated into two clusters with a mixed distribution of serotypes. For serotype O3:K6, 62 out of 63 strains were present in Cluster I, while its pandemic serovariants, such as O4:K68 and O1:KUT, were present in both clusters. Two isolates of O1:K25 were present in Cluster I. For serotype O4:K8, 32 out of 36 strains were present in Cluster II, in addition to 14 other strains of the same group. All isolates of O8:K41 were present in the same group as O4:K8. Some rare serotypes, such as O1:K36, were present in both clusters.

### Phyloproteomic analysis of O4:K8, O3:K6 and its three main serovariants O4:K68, O1:K25 and O1:KUT

**Fig. 1:**
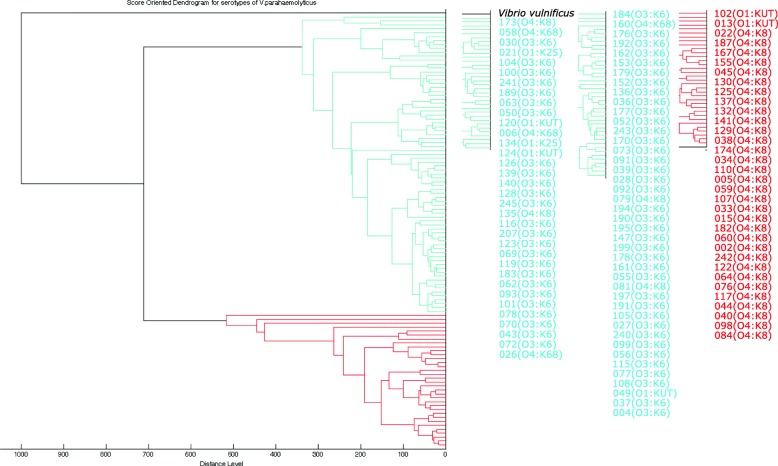
MALDI-TOF MS-based dendrogram of 110 strains belonging to O3:K6, O4:K8, O4:K68, O1:K25 and O1: KUT

Figure 1 shows the proteomic cluster analysis of 110 strains belonging to five serotypes (O4:K8, O3:K6, O4:K68, O1:K25 and O1:KUT). All isolates of O3:K6 and its serovariants O4:K68 and O1:K25 were present in Cluster I. However, two out of five strains of O1:KUT were separated into Cluster II. For serotype O4:K8, four out of 36 strains were clustered into the same group as O3:K6, and the remaining 32 strains were present in Cluster II.

**Fig. 2:**
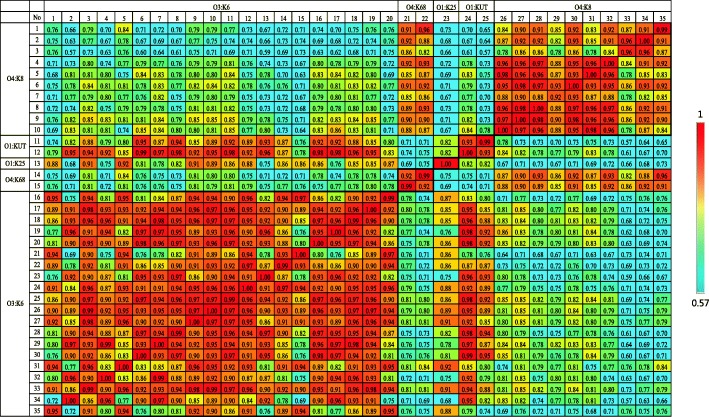
Composite Correlation Index (CCI) matrix of 35 strains (20 of O3:K6, ten of O4:K8, two of O1: KUT, two of O4:K68 and one of O1:K25) as calculated using Biotyper RTC software. The CCI values were extracted and the displayed image was redrawn using the conditional formatting option in MS Excel. A CCI value approaching 1 indicates congruence among the measured spectra sets and a CCI value of 0 represents completely different spectra

Principal component analysis (PCA) was used to confirm the difference between O4:K8 and the four other serotypes (Additional file 2: Figure S2). Two distinct clusters indicate that there is no obvious correlation between the O4:K8 strains, the O3:K6 strains and the three other pandemic serotypes. These results were also confirmed by composite correlation index (CCI) analysis. A CCI value of 1 represents the highest possible correlation, while a value of 0 represents no correlation. A CCI matrix of representative mass spectra was obtained from all 110 strains belonging to five pandemic serotypes (Additional file 3: Figure S3). A simplified matrix was also obtained using fewer strains and this matrix depicts the differences between the groups clearly (Fig. 2). Most spectra belonging to the O4:K8 serotype were dissimilar to the spectra belonging to O3:K6 and the three other pandemic serotypes; these were denoted as “cold” (green to dark blue). The low CCI values that result from the comparison of O4:K8 with O3:K6, O4:K68, O1:K25 and O:KUT indicate that the discrimination of O4:K8 using spectral fingerprinting is possible.

### Potential peaks contributing to the differentiation of O4:K8 from O3:K6 and its three main pandemic serovariants

**Fig. 3:**
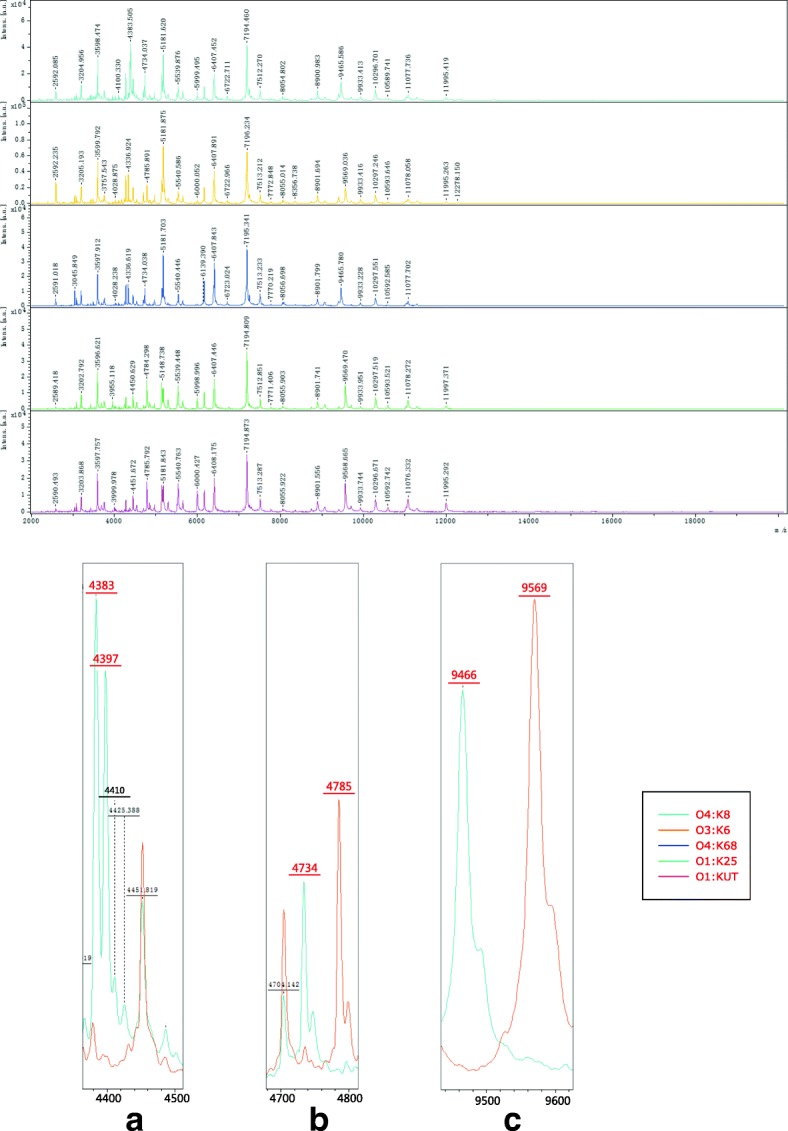
MALDI-TOF MS spectra of five isolates belonging to five serotypes (O4:K8, O3:K6, O4:K68, O1:K25 and O1:KUT) displayed in FlexAnalysis: (**a**) Peaks at *m/z* 4383 and 4397 of O4:K8 compared with O3:K6; (**b**) Different distribution of two peaks at *m/z* 4734 and 4785 belonging to O4:K8 and O3:K6, respectively; (**c**) Comparison of two peaks at *m/z* 9466 and 9569 belonging to O4:K8 and O3:K6, respectively

Potential peaks contributing to the differentiation of O4:K8 from four other serotypes (O3:K6, O4:K68, O1:K25 and O1:KUT) were identified using ClinProTools 3.0. The performances of three different models (the Genetic Algorithm, the Supervised Neural Network, and the QuickClassifier Algorithm) were evaluated. The Genetic Algorithm produced the best results (99.91% recognition capability and 99.04% cross validation) (Table 3). In addition, the differences between the peaks of O4:K8, O3:K6 and its serovariants (O4:K68, O1:K25, and O1:KUT) at *m/z* 4383, 4397, 4734, 4785, 9466 and 9569 could be observed in the mass spectra (Fig. 3).

**Table 3:** The performance of models generated by ClinProTools for the differentiation of O4:K8 from O3:K6 as well as its three serovariants (O4:K68, O1:K25, and O1:KUT)

Target serotype	Control serotype	Classification algorithm	Peaks used in the model	Cross validation (%)	Recognition capability (%)
				Overall	Overall
O4:K8 (*n* = 36)	O3:K6(*n* = 63) O4:K68(n = 4) O1:K25(*n* = 2) O1:KUT(*n* = 5)	GA	4798.1, 8357.42, 8090.06, 6395.04, 8173.94	99.1	99.82
		QC	3512.86,3557.98, 3740.45, 4383.85, 4397.89, 4486.14, 4734.61, 4785.89, 7051.75, 7405.26, 8090.06, 9466.35, 9569.91	94.0	93.8
		SNN	9466.35, 4486.14	92.7	93.11

*GA* genetic algorithm, *QC* QuickClassifier Algorithm, *SNN* Supervised Neural Network Algorithm

**Fig. 4:**
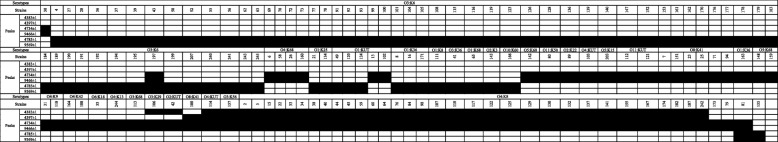
The distribution of peaks at *m/z* 4383, 4397, 4734, 4785, 9466 and 9569 in 146 strains belonging to 23 serotypes are shown in the graph. The black squares highlight the presence of peaks in three replicated spectra of a strain

The presence or absence of the peaks at *m/z* 4383, 4397, 4734, 4785, 9466 and 9569 in 146 strains was also recorded (Fig. 4). Among O4:K8 isolates, 35 out of 36 strains contained peaks at *m/z* 4734 and 9466, and 32 out of 36 strains contained peaks at *m/z* 4383, 4397, 4734 and 9466 simultaneously. Only two strains of O4:K8 possessed peaks at *m/z* 4785 and 9569. Only five out of the 110 non-O4:K8 strains contained peaks at *m/z* 4383 and/or 4397; none of the strains belonging to the four pandemic serotypes (O3:K6, O4:K68, O1:25 and O1:KUT) contained these two peaks. 61 out of 63 isolates of O3:K6 contained peaks at *m/z* 4785 and 9569. Both isolates of O1:K25 contained two peaks, at *m/z* 4785 and 9569. But for O4:K68, both of them contained two peaks at *m/z* 4734 and 9466. Two out of five O1:KUT isolates contained peaks at *m/z* 4734 and 9466, while the other isolates contained peaks at *m/z* 4785 and 9569.

## Discussion

The 146 strains in this study were divided into 23 serotypes; O3:K6 was the most common serotype, followed by O4:K8 and then O8:K41. Notably, isolates of O3:K6 and O4:K8 make up a large proportion of all the analyzed strains. The distribution of serotypes in Zhejiang province was slightly different from that of Jiangsu province, where O3:K6, O5:K17 and O1:KUT were the three major serotypes [26].

Since 1996, serotype O3:K6 with specific genetic markers (with *tdh* and *toxRS/new* genes, and with or without *orf8* genes) has emerged as a major serotype, causing worldwide outbreaks, including outbreaks in China. Infections associated with serotype O4:K8 have occurred in Peru, where this serotype remains predominant [27]. In China, the number of infections associated with O4:K8 has recently increased. This serotype has emerged as a distinct group that is almost different from O3:K6. Nearly 21 serotypes, including O4:K8 [28], are recognized as serovariants of O3:K6. These serotypes share identical genotypes and molecular profiles [11] and are therefore called “O3K6 clones” or “pandemic strains”. The majority of these have established an ecological niche in Asia [29]. O4:K68, O1:K25 and O1:KUT, which are clonally related to O3:K6, are responsible for pandemic outbreaks [30]. O4:K8, a serovariant of O3:K6, formed a potential predominant clone in South China. Cluster analysis of O4:K8, O3:K6 and O1:KUT showed that all strains of O4:K8 were clustered into one group, while O3:K6 and O1:KUT, isolated from different countries and regions, were clustered into another group [31]. Notably, we found a similar cluster for O4:K8 using proteomic analysis with Biotyper 4.0, while O3:K6, O4:K68, O1:K25 and O1:KUT were clustered into another group. This confirms that O4:K8 represents a threat as a thriving serotype of *V. parahaemolyticus* infection, which is distinct from O3:K6. Four out of 36 O4:K8 strains were clustered into the same group as O3:K6. This is explained by the fact that O4:K8 is designated as a serovariant of O3:K6. Furthermore, there is a possibility that these four strains had not evolved into a specific group that is different from O3:K6.

Identification of strains using the original commercial database together with our in-house database resulted in a high log score. This indicates that a sufficient number of strains are needed in the database for reliable identification. Biomarker based databases have been illustrated to an optimism method which superior to pattern recognition based databases and Vitek 2™ for the identification of Gram negative bacteria [32]. Several studies have also used ribosomal biomarkers corresponding to sequence types and/or clonal complexes to subtype *Neisseria meningitides* [33]. A recent study also successfully identified *Clostridium difficile* genotype ST37 by MALDI-TOF MS, and further characterized two peaks at *m/z* 3242 and 3286 that were specific for ST37 [34]. In the current study, peaks at *m/z* 4383 and 4397 appear to be specific for serotype O4:K8, allowing for the discrimination of O4:K8 from O3:K6 and its three other serovariants. Characterization of the two potential biomarkers at *m/z* 4383 and 4397 is in progress. The inclusion of isolates from different regions should confirm the ability of MALDI-TOF MS to discriminate O4:K8 from other pandemic serotypes. Further attention should be given to infections associated with serotype O4:K8.

## Conclusion

This study supports the hypothesis that O4:K8, which thrives as a potential predominant clone, is distinct from O3:K6 and its three main serovariants at the protein level. The use of MALDI-TOF MS in a study of this nature has not been reported before. Furthermore, peaks in the mass spectra at *m/z* 4383 and 4397 were determined to be specific for O4:K8 over four pandemic serotypes. However, we cannot conclude that MALDI-TOF MS can be used for the serotyping of *V. parahaemolyticus*. The taxonomic resolution of the MALDI-TOF MS technique is occasionally overestimated, and caution must be exercised when using MALDI-TOF MS to distinguish the serovariants of *V. parahaemolyticus* and other species.

## Materials and methods

### Reagents

Ethanol, formic acid, acetonitrile (AN), trifluoroacetic acid (TFA), and *α*-cyano-4-hydroxycinnamic acid (CHCA) were purchased from Fluka, Germany.

### Bacterial strains

A total of 146 strains of *V. parahaemolyticus*, including 23 serotypes, were tested in this study. All strains were isolated in Zhejiang province, China, and the clinical strains were collected as part of standard care. Whole isolates were grown on TCBS, and then further confirmed by *toxR*-target PCR amplification. Conventional serotyping was performed by slide agglutination with a *V. parahaemolyticus* antiserum (Denka Seiken, Tokyo, Japan).

### Sample preparation

Bacterial strains were grown overnight on heart infusion (HI) agar. The cultures were harvested and subjected to ethanol-formic acid extraction as previously described [35]. 1 μl of each supernatant was spotted on to a Bruker target plate, and each strain was spotted in 5 replicates. Each spot was overlaid with 2 μl of 10 mg/ml CHCA in 2.5% aqueous TFA and allowed to dry at room temperature.

### MALDI-TOF MS parameters and spectrum generation

The spectra were obtained using a microflex LT bench top mass spectrometer (Bruker Daltonik GmbH, Bremen, Germany) using a 20 Hz nitrogen laser and the following parameters: parameter settings: ion source 1 (IS1), 20 kV; IS2, 18.5 kV; lens, 8.5 kV; detector gain, 2650 V; and gating, none. A total of 300 shots per composite mass spectrum were recorded with the positive linear mode in a spectrum range of *m/z* 2000–20,000. The instrument was externally calibrated with a bacterial test standard (BTS, Bruker Daltonics). Theoretical and measured masses matched within 300 ppm. After calibrating manually, 20 independent spectra from different laser aiming spots of one sample were obtained.

### MSP library construction

Thirty five isolates were randomly selected for use in the in-house database (Table 2), all of which belonged to O3:K6, O4:K8, O1:K5, O1:KUT or O4:K68. Twenty spectra, representing five technical replicates, were collected for every strain/isolate. The remaining 75 strains were analyzed for evaluation of this database. The spectra preprocessing parameters included mass adjustment (lower bound = 3000, upper bound = 15,000, resolution = 1, data reduction factor = 10), smoothing (Savitzky-Golay algorithm with a frame size 25 Da), baseline correction (multipolygon with number of run 1), normalization (maximum norm, the spectrum was normalized to a maximum peak value of 1), and peak detection (spectra differentiation with maximum peaks of 100 and threshold of 0.001). Main spectrum profiles (MSPs) were created for each strain using the following parameters: maximum mass error of each single spectrum = 6000, mass error for the MSP = 200, peak frequency minimum = 5%, maximum peak number = 100.

Log (score) values ≥2.0 were considered for species-level identification and log (score) values < 2 and ≥ 1.7 for were considered for genus-level identification. Results based on log (score) values < 1.7 were rated as unidentifiable. For species identification, raw spectra of 146 isolates were imported into the BioTyper software and matched against the commercial database (with default parameter settings). Comparisons between the commercial database and the expanded database after the introduction of the in-house database were also conducted. Spectra of the remaining 75 strains, which belonged to pandemic serotypes and were not used for database construction, were loaded into MALDI Biotyper Compass Explorer 4 and matched against the commercial database with 8223 MSPs entries and the expanded database after the introduction of the in-house database.

### Phyloproteomic analysis of *V. parahaemolyticus*

To further evaluate the proteomic relatedness of different serotypes, dendregram analyses for 146 strains of 23 serotypes and 110 strains of five serotypes (O4:K8, O3:K6, O4:K68, O1:K25 and O1:KUT) were performed using BioTyper 4 software (Bruker Daltonics). These analyses were based on MSPs with the default parameters. The distance measurement was set to “correlation” and the linkage algorithm was set to “average”, while *Vibrio vulnific* ATCC27562 was included as an out-group.

The differences between O4:K8 and the four other main pandemic serotypes (O3:K6, O1:K25, O1:KUT and O4:K68) were further analyzed using PCA and CCI [36]. Dendrograms obtained in PCA represented the closeness of each spectrum to one another [37]. The default parameters of PCA clustering were set to “hierarchical”, the distance measurement was set to “correlation” and the linkage algorithm was set to “average”. To further evaluate the spectral variation (similarity) between the spectra sets acquired from these five main pandemic serotypes, the CCI was calculated using MALDI Biotyper Compass Explorer 4. All measured spectra were loaded and the CCI was calculated using the following settings: mass lower bound = 3000, mass upper bound = 12000, resolutio*n* = 4 and CCI parameter interval = 8. A CCI value of 1 indicates complete correlation between the spectra, while a value of 0 indicates no correlation. The CCI values were extracted and the displayed image was redrawn using the conditional formatting option in MS Excel.

### Identification of potential peaks that contribute to the differentiation of O4:K8 from the other four serotypes using ClinProTools

We generated three models to identify potential biomarkers that differentiate O4:K8 from the four other main serotypes (O3:K6, O4:K68, O1:K25 and O1:KUT) using ClinProTools. We used three algorithms: the Genetic Algorithm, the Supervised Neural Network, and the QuickClassifier Algorithm. All isolates of O4:K8 were used as a target group, while the other strains were used as a control group. Mass data files of these two groups were transferred into ClinProTools and recalibrated with the default parameters of 1000 ppm maximal peak shift. Several mass peaks (*m/z*) with significant differences were selected for classification by the model.

## Additional files

Additional file 1: **Figure S1.** MALDI-TOF MS-based dendrogram of 146 strains belonging to 23 serotypes. The strains were separated into two clusters with a mixed distribution of serotypes. (PDF 164 kb)

Additional file 2: **Figure S2.** Principal component analysis of 110 strains belonging to five main pandemic serotypes. 110 strains were separated into two main clusters. Groug I contained all O1:K25 strains, 62 out of 63 O3:K6 strains, four out of five O1:KUT strains, half of O4:K68 strains and three out of five O4:K8 isolates. Group II were composed of 33 out of 36 O4:K8 strains, two of O4:K68, and one of O1:KUT and O3:K6, respectively. Strains included in each cluster were almost similar to those in MSP dendrogram analysis. (PDF 429 kb)

Additional file 3: **Figure S3.** Composite Correlation Index (CCI) matrix of 110 strains (O3:K6, O4:K8, O1:K25, O1:KUT and O4:K68) as calculated using Biotyper RTC software. The CCI values were extracted and the displayed image was redrawn using the conditional formatting option in MS Excel. A CCI value approaching 1 indicates congruence among the measured spectra sets and a CCI value of 0 represents completely different spectra. (PDF 139 kb)

## Abbreviations

AN: Acetonitrile
BTS: Bacterial test standard
CCI: Composite correlation index
CHCA: *α*-cyano-4-hydroxycinnamic acid
GS-PCR: Group specific polymerase chain reaction
MALDI-TOF MS: Matrix-assisted laser desorption/ionization time-of-flight mass spectrometry
MSP: Main spectrum profile
PCA: Principal component analysis
PCR: Polymerase chain reaction
T3SS: Type III secretion systems
TDH: Thermostable direct haemolysin
TFA: Trifluoroacetic acid
TRH: TDH-related haemolysin
