# Supplementary material for: MALDI-TOF mass spectrometry-based serotyping of V. parahaemolyticus isolated from the Zhejiang province of China
Source: BMC Microbiol. 2018 Nov 13;18:185. doi: 10.1186/s12866-018-1328-z (PMC6234682; doi:10.1186/s12866-018-1328-z)
Supplement: Supplementary file 1 — Figure S1. MALDI-TOF MS-based dendrogram of 146 strains belonging to 23 serotypes. The strains were separated into two clusters with a mixed distribution of serotypes. (PDF 164 kb) [file 12866_2018_1328_MOESM1_ESM.pdf]

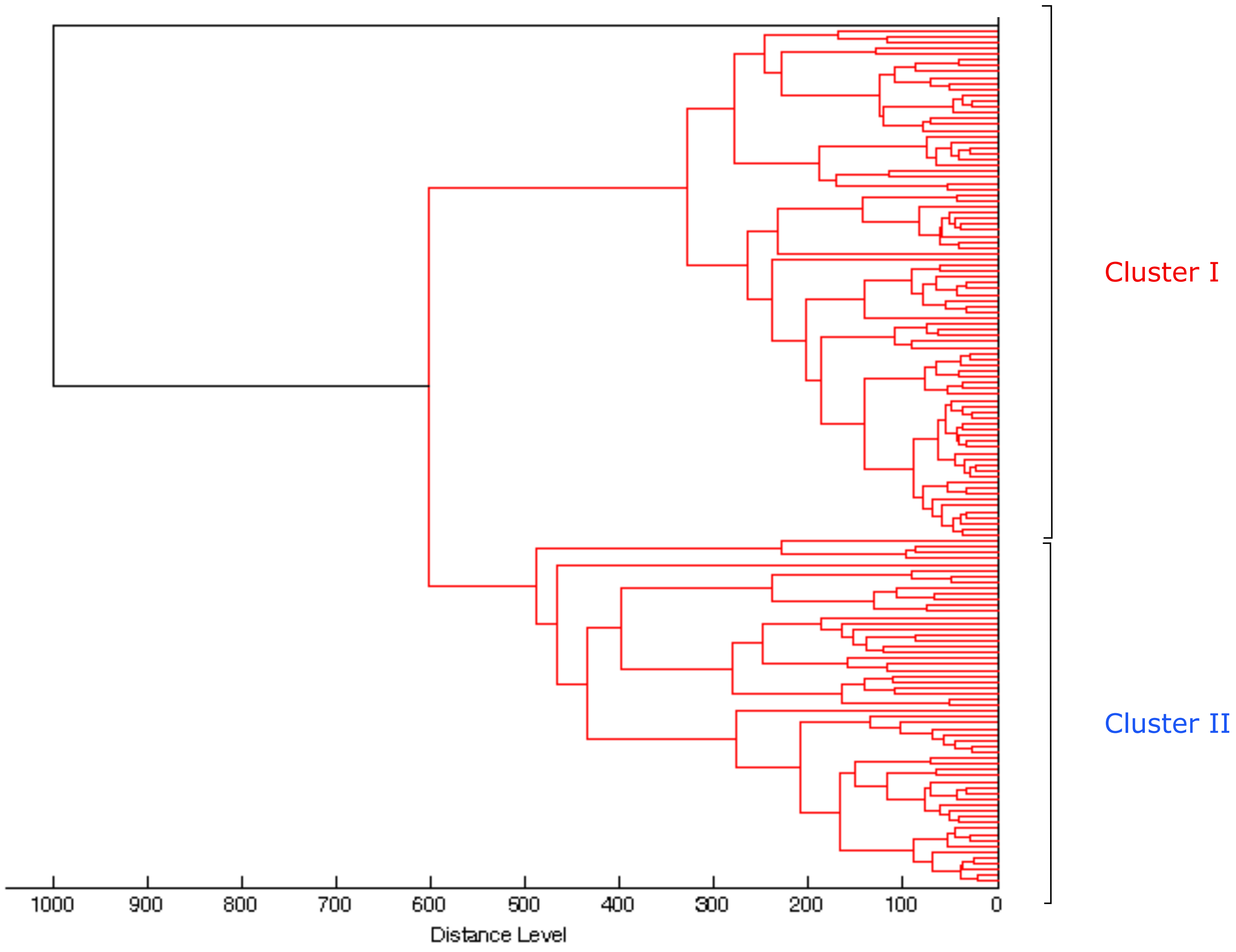

Cluster I			Cluster II	
106(O3:K29)	171(O1:K36)	091(O3:K6)	102(O1:KUT)	130(O4:K8)
080(O11:K50)	140(O3:K6)	039(O3:K6)	094(O8:K41)	125(O4:K8)
058(O4:K68)	139(O3:K6)	028(O3:K6)	025(O8:K41)	137(O4:K8)
166(O10:K60)	245(O3:K6)	194(O3:K6)	007(O8:K41)	141(O4:K8)
065(O1:K68)	128(O3:K6)	190(O3:K6)	013(O1:KUT)	132(O4:K8)
207(O3:K6)	126(O3:K6)	195(O3:K6)	167(O4:K8)	129(O4:K8)
123(O3:K6)	135(O4:K8)	147(O3:K6)	157(O3:K56)	038(O4:K8)
069(O3:K6)	116(O3:K6)	199(O3:K6)	155(O4:K8)	174(O4:K8)
119(O3:K6)	041(O3:K36)	178(O3:K6)	159(O5:K68)	034(O4:K8)
183(O3:K6)	184(O3:K6)	161(O3:K6)	160(O4:K68)	110(O4:K8)
062(O3:K6)	192(O3:K6)	197(O3:K6)	148(O5:K68)	005(O4:K8)
093(O3:K6)	143(O2:K3)	081(O4:K8)	164(O4:K42)	059(O4:K8)
101(O3:K6)	176(O3:K6)	092(O3:K6)	045(O4:K8)	107(O4:K8)
078(O3:K6)	162(O3:K6)	055(O3:K6)	173(O4:K8)	033(O4:K8)
070(O3:K6)	153(O3:K6)	191(O3:K6)	163(O1:K36)	015(O4:K8)
072(O3:K6)	152(O3:K6)	105(O3:K6)	112(O11:KUT)	182(O4:K8)
043(O3:K6)	179(O3:K6)	027(O3:K6)	244(O4:K13)	060(O4:K8)
026(O3:K6)	136(O3:K6)	240(O3:K6)	113(O3:K68)	180(O8:K41)
104(O3:K6)	036(O3:K6)	099(O3:K6)	188(O4:K42)	002(O4:K8)
100(O3:K6)	008(O1:K36)	056(O3:K6)	035(O6:K18)	242(O4:K8)
241(O3:K6)	203(O5:K15)	016(O1:K36)	121(O11:KUT)	122(O4:K8)
189(O3:K6)	142(O5:K68)	115(O3:K6)	042(O2:KUT)	114(O4:KUT)
063(O3:K6)	089(O2:K22)	108(O3:K6)	022(O4:K8)	064(O4:K8)
050(O3:K6)	111(O1:K8)	049(O1:KUT)	118(O4:K9)	076(O4:K8)
120(O1:KUT)	079(O4:K8)	037(O3:K6)	031(O4:K9)	117(O4:K8)
021(O1:K25)	243(O3:K6)	077(O3:K6)	071(O8:K41)	044(O4:K8)
103(O4:KUT)	170(O3:K6)	004(O3:K6)	030(O3:K6)	040(O4:K8)
006(O4:K68)	073(O3:K6)		023(O8:K41)	098(O4:K8)
134(O1:K25)	177(O3:K6)		151(O8:K41)	084(O4:K8)
124(O1:KUT)	052(O3:K6)		187(O4:K8)	
