# Supplementary material for: MALDI-TOF mass spectrometry-based serotyping of V. parahaemolyticus isolated from the Zhejiang province of China
Source: BMC Microbiol. 2018 Nov 13;18:185. doi: 10.1186/s12866-018-1328-z (PMC6234682; doi:10.1186/s12866-018-1328-z)
Supplement: Supplementary file 2 — Figure S2. Principal component analysis of 110 strains belonging to five main pandemic serotypes. 110 strains were separated into two main clusters. Groug I contained all O1:K25 strains, 62 out of 63 O3:K6 strains, four out of five O1:KUT strains, half of O4:K68 strains and three out of five O4:K8 isolates. Group II were composed of 33 out of 36 O4:K8 strains, two of O4:K68, and one of O1:KUT and O3:K6, respectively. Strains included in each cluster were almost similar to those in MSP dendrogram analysis. (PDF 429 kb) [file 12866_2018_1328_MOESM2_ESM.pdf]

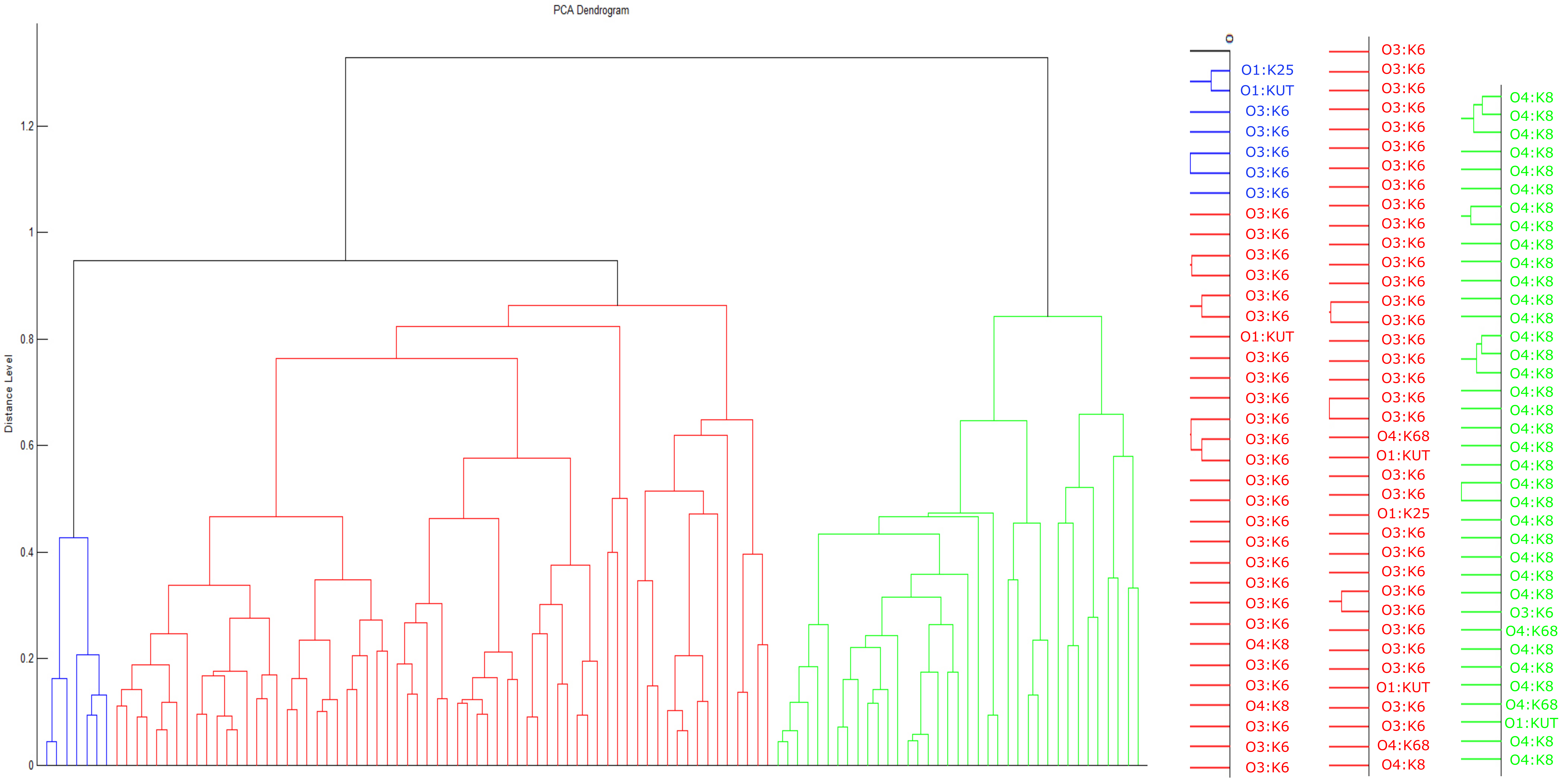

PCA Dendrogram
Distance Level
0
0.2
0.4
0.6
0.8
1
1.2
0
O1:K25
O1:KUT
O3:K6
O3:K6
O3:K6
O3:K6
O3:K6
O3:K6
O3:K6
O3:K6
O3:K6
O3:K6
O3:K6
O1:KUT
O3:K6
O3:K6
O3:K6
O3:K6
O3:K6
O3:K6
O3:K6
O3:K6
O3:K6
O3:K6
O3:K6
O3:K6
O3:K6
O3:K6
O4:K8
O3:K6
O3:K6
O4:K8
O3:K6
O3:K6
O3:K6
O3:K6
O3:K6
O3:K6
O3:K6
O3:K6
O3:K6
O3:K6
O3:K6
O3:K6
O3:K6
O3:K6
O3:K6
O3:K6
O3:K6
O3:K6
O3:K6
O3:K6
O3:K6
O3:K6
O3:K6
O3:K6
O4:K68
O1:KUT
O3:K6
O3:K6
O1:K25
O3:K6
O3:K6
O3:K6
O3:K6
O3:K6
O3:K6
O3:K6
O3:K6
O1:KUT
O3:K6
O3:K6
O4:K68
O4:K8
O4:K8
O4:K8
O4:K8
O4:K8
O4:K8
O4:K8
O4:K8
O4:K8
O4:K8
O4:K8
O4:K8
O4:K8
O4:K8
O4:K8
O4:K8
O4:K8
O4:K8
O4:K8
O4:K8
O4:K8
O4:K8
O4:K8
O4:K8
O4:K8
O4:K8
O4:K8
O4:K8
O4:K8
O3:K6
O4:K68
O4:K8
O4:K8
O4:K8
O4:K68
O1:KUT
O4:K8
O4:K8
